# Protocol for a randomized controlled trial of the Breaking Free Online Health and Justice program for substance misuse in prison settings

**DOI:** 10.1186/s40352-018-0078-1

**Published:** 2018-11-03

**Authors:** Sarah Elison-Davies, Glyn Davies, Jonathan Ward, Stephanie Dugdale, Samantha Weston, Andrew Jones, Michelle Brides, John Weekes

**Affiliations:** 1 Breaking Free Group, Manchester Science Park, Manchester, M15 6LR UK; 2 University of Keele, School of Social Science and Public Policy, Staffordshire, ST5 5BG UK; 3 University of Manchester, Centre for Epidemiology, Manchester, M13 6PL UK; 4 Spectrum Community Health, HMP Preston, 2 Ribbleton Lane, Preston, PR1 5AB UK; 5 Waypoint Centre for Mental Health Care, Penetanguishene, ON L9M 1G3 Canada; 6 Department of Psychology, Carleton University, Ottawa, ON K1S 5B6 Canada; 7 Michael G. DeGroote School of Medicine, McMaster University, Hamilton, ON L8S 4L8 Canada

**Keywords:** Substance misuse, Alcohol, Drugs, Computer-assisted therapy, Recovery, Prisons, Criminal justice

## Abstract

**Background:**

Substance misuse, including problematic drug and alcohol use, are significant issues in society that can have multiple detrimental effects. Many people access support for their substance misuse during prison sentences, due to the associations between substance misuse and offending, and the high proportion of the prison population who have drug and alcohol issues. Breaking Free Online Health and Justice is a computer-assisted therapy program that has been developed to support substance-involved offenders to address their substance misuse and associated offending within prison settings.

**Methods:**

This will be a parallel-group randomized controlled trial of 4-week Breaking Free Online Health and Justice program as an adjunct to standard treatment for substance misuse, in comparison to standard treatment only, in a male Category D open prison. Interventional and control groups will be compared in terms of the changes in their scores on multiple measures from baseline to post-treatment assessment at 4-weeks, and then 3- and 6-months follow-up. Participants will be adult male offenders serving sentences in prison in England who have demonstrable difficulties with drugs and/or alcohol for at least the past 12-months. The primary outcome measure will be self-reported substance misuse, with secondary outcomes being standardized psychometric assessments of substance dependence, mental health, biopsychosocial functioning, quality of life and post-release offending. Other secondary measures will include frequency of completion of specific intervention strategies in the program.

**Discussion:**

This study will examine whether Breaking Free Online Health and Justice as an adjunct to standard substance misuse interventions in prisons, improves outcomes for substance-involved offenders receiving interventions in custodial settings. Findings from the study will be used to inform further developments of the program and potential improvements to custodial treatment.

**Trials registration:**

ISRCTN09846981.

**Electronic supplementary material:**

The online version of this article (10.1186/s40352-018-0078-1) contains supplementary material, which is available to authorized users.

## Background

Substance misuse, including problematic drug and alcohol use, are significant issues in society that can have multiple detrimental effects. Substance misuse is implicated in a number of criminal offences, including acquisitive crime (Comiskey et al. 2012; Hayhurst et al. 2013), anti-social and violent behavior (Boden et al. 2012; Lundholm et al. 2013), domestic and intimate partner violence (Stuart et al. 2008; Wilson et al. 2017) and child neglect Solis et al. (2012). Links between substance use and criminal behavior are identified within the research literature (Bennett et al. 2008; Hough 2002; Schroeder et al. 2007). Levels of crime reported by substance users during periods of use (Ball et al. 1983; Bennett and Holloway 2009; Bennett et al. 2008; Best et al. 2001; Goldstein 1985; Gossop et al. 2000; Inciardi 1979; McGlothlin et al. 1978), and the high proportion of the prison population who are substance misusers (Budd et al. 2005; Jones et al. 2007; Phillips 2000; Young et al. 2011) all indicate that substance misuse and offending often co-occur and that substance misuse is a primary “criminogenic” factor (Weekes et al. 1999).

In the 2016 Crime Survey for England and Wales, 8.4% of 16–59 year old participants living in the UK reported using an illicit drug within the last 12-months, which, if representative, would extrapolate to approximately 2.7 million people (Home Office 2016). The economic costs to society of substance misuse are substantial, with problematic alcohol misuse alone being estimated to have cost £ 47 billion in 2016 (PHE 2016). Recent data reported by Public Health England from the National Drug Treatment Monitoring System demonstrate that overall, 279,793 adults were in contact with drug and alcohol services between 2016 and 2017 (PHE 2017), with 26% of those receiving treatment for opiate dependence being referred into treatment by criminal justice services. Furthermore, up to 48% of those seeking treatment for both opiate and ‘novel psychoactive substance’ dependence were referred via the criminal justice system (PHE 2017). And additionally, a recent systematic review which included studies from multiple countries, found that both alcohol use disorder and substance use disorder are highly prevalent amongst the prison population, with pooled prevalence estimates of each being 24% and 51% respectively Fazel et al. (2017).

Given the significant associations between substance use and offending, it seems intuitive that if any intervention for substance-involved offenders is to be effective, it needs to address not only the substance use but also the offending behavior that may be associated with it Elison et al. (2017a). In order to meet this requirement, Breaking Free Online (BFO) Health and Justice, a computer-assisted therapy (CAT) program designed to address both substance misuse and offending behaviors simultaneously, has been developed. Such CAT approaches have the potential to widen access to evidence-based treatment for substance misusing individuals as they can be delivered at scale, and because intervention content is delivered via a computer in a highly standardized way, CAT can enhance treatment fidelity and thus treatment effectiveness (Bickel et al. 2008; Moore et al. 2011).

This criminal-justice specific version of BFO has been developed via modification of a version of the program that has been delivered in community-based substance misuse treatment settings for the past eight-years. Published research informed by guidance by the UK Medical Research Council (MRC) around the development and evaluation of complex interventions (Craig et al. 2008; Moore et al. 2015) has examined the evidence-base underpinning the clinical content of BFO (Dugdale et al. 2016b), and barriers and facilitators of the implementation of the program in real-world treatment settings (Dugdale et al. 2017; Dugdale et al. 2016a, b; Elison et al. 2014a, b; Ward et al. 2017). Research examining the effectiveness of the program (Elison et al. 2015a, b; Elison et al. 2014a, b; Elison et al. 2017d) has demonstrated significant reductions in substance dependence and use, and significant improvements in mental health and broader psychosocial functioning. Examination of the mechanisms of action of BFO has demonstrated that users follow tailoring advice provided by the program, that the program exhibits a ‘dose-response’, and that completion of cognitive restructuring strategies in the program underpins changes to broader biopsychosocial functioning (Elison et al. 2017c).

Since 2015 BFO has been available in prisons in addition to community settings via the ‘Virtual Campus’ (VC), the UK prisons IT infrastructure that allows offenders to access a limited range of online programs to support their education, training and employment. However, BFO has become the first *healthcare* program to be included on VC, and the first digital intervention for offenders to be accredited by the UK Ministry of Justice, Correctional Services Advice and Accreditation Panel. Mixed-methods research conducted by the authors explored both the barriers and facilitators of implementation of BFO in prison settings (Elison et al. 2015c; Elison et al. 2016b), and examined clinical outcomes for offenders accessing the program as part of the ‘Gateways’ through-care initiative (Elison et al. 2015c; Davies et al. 2017), which aimed to support substance-involved offenders as they transition back to the community.

Qualitative interview data from 16 offenders engaging with BFO and 10 members of prison staff supporting them suggested that both offenders and staff were able to overcome initial anxieties about using digital technology. Offenders reported the program supported them to develop coping skills to enable them to remain abstinent from using drugs and alcohol, and therefore reduce their chances of reoffending when they were released (Elison et al. 2015c). Staff reported that they felt the program provided an opportunity for offenders to access an evidence-based intervention to allow them to work on their drug and alcohol difficulties, and also provided an opportunity to use the VC in a novel way to further support offender rehabilitation (Davies et al. 2017). Analyses of quantitative clinical outcomes from a sample of 151 male offenders accessing BFO before being released from prison (Davies et al. 2017; Elison et al., 2015c), demonstrated significant reductions in alcohol and drug dependence and consumption, significant improvements in quality of life, and significant improvements in multiple aspects of broader biopsychosocial functioning. However, only within-subject analyses were conducted in this research, with no ‘standard treatment’ control group having been included in the research conducted to date.

## Method

### Aims

This study will evaluate, via a randomized controlled trial (RCT) methodology, the efficacy of BFO as a supplement to standard treatment, within a criminal justice setting. The principle aim of this study is therefore to determine the effectiveness of BFO delivered alongside standard treatment, in comparison to standard treatment only, in reducing alcohol and drug consumption and dependence, and any possible impact on mental health and broader biopsychosocial functioning. It is anticipated that delivery of BFO alongside standard treatment should confer some added benefits to participants engaging with this novel intervention, when compared to participants engaging with standard treatment only. This means there may be some post-treatment differences between the two study groups in terms of substance-related outcomes and broader biopsychosocial functioning.

### Design

This will be a randomized, parallel-group longitudinal comparison study of 4-week periods of either i) BFO plus standard treatment, or ii) standard treatment only, using intention to treat (ITT) analyses to examine outcomes.

### Setting

The study will be conducted in an adult male prison in North-West England, UK, where the BFO program is not currently delivered as a standard treatment. This prison is a ‘Category D’ open prison where offenders are, subject to approval, provided with ‘Release on Temporary License’ (ROTL) where they are granted release to work in the community or have ‘home leave’. This prison is a resettlement prison which has an operational capacity of just over 600 male offenders, approximately a quarter of whom are either on life sentences or subject to indeterminate sentences. Approximately, 75% of the men in the prison are serving sentences of 4-years or longer at any one time. Around three-quarters of the men in the prison are over the age of 30 years, and around 40% have identified substance misuse difficulties. The prison places a strong emphasis on rehabilitation and community reintegration, running a range of vocational training courses, alongside initiatives to support the men serving sentences in the prison to maintain and enhance the relationships they have with their families.

This category of prison has been chosen for the study because, although even the very highest security prisons in the UK have significant issues with drug and alcohol use, offenders in a Category D prison may potentially have the most opportunity to use substances, as they spend some of their time in the community. Most participants may be on ROTL during the study, including resettlement day release, and resettlement overnight release, so it is more likely that outcomes related to substance use will be an artifact of treatment effects, rather than lack of opportunity to use substances due to incarceration in a highly secure environment. Although it is anticipated that most, if not all, participants will be receiving ROTL during the study, the ROTL status of each participant will be recorded during their first treatment session and this will be taken into account during data analyses, if there are a significant number of participants not provided with ROTL during the study. Participants will be recruited from standard alcohol and drug misuse services in the prison, which are delivered in coordination with Her Majesty’s Prison and Probation Service (HMPPS).

### Participants

Participants included in the study will be offenders currently serving a prison sentence aged 18 to 65 years with problem alcohol and/or drug use of duration of 12-months or longer. This period of time is in line with DSM-V criteria for substance related disorders (American Psychiatric Association 2000). It is estimated that a total of 240 participants will need to be recruited and screened in order to obtain a sample of 120 evaluable participants (see ‘Power Calculation’).

- Inclusion criteria
  1. Male offenders currently serving a prison sentence with problem alcohol and/or drug use aged 18 to 65 years.
  2. Willing and able to give informed consent for participation in the study.
  3. Has at least 3-months left to serve of their sentence at the prison acting as the research site, at the time they are recruited to the study.
  4. Problem alcohol and/or drug use present for at least 12-months prior to current prison sentence.
  5. Willing to follow treatment for problem alcohol and/or drug use for 4-weeks.
  6. Willing to provide outcome measures at 3- and 6-months follow-up.
  7. Concomitant alcohol and drug/s use permitted, as well as any prescribed medication.
- Exclusion Criteria
  1. Participation in any other alcohol and/or drug related clinical studies.
  2. Individuals detained under the Mental Health Act.
  3. Individuals with a known and diagnosed intellectual or developmental disability.
  4. Non-English speaking offenders (study information material and program only produced in English).

### Interventions

#### Breaking free online health and justice

BFO is an online treatment program for substance-involved offenders. Clinical content of BFO has been informed by the available evidence-base around effective biopsychosocial and behavioral intervention approaches for addressing drug and alcohol misuse (National Treatment Agency for Substance Misuse 2006a, b; NICE 2007, 2011, 2012), including cognitive-behavioral principles (Beck 1993; Beck et al. 2011), and other approaches including mindfulness-based relapse prevention (Marlatt et al. 2010; Marlatt and Donovan 2005).

**Fig. 1:**
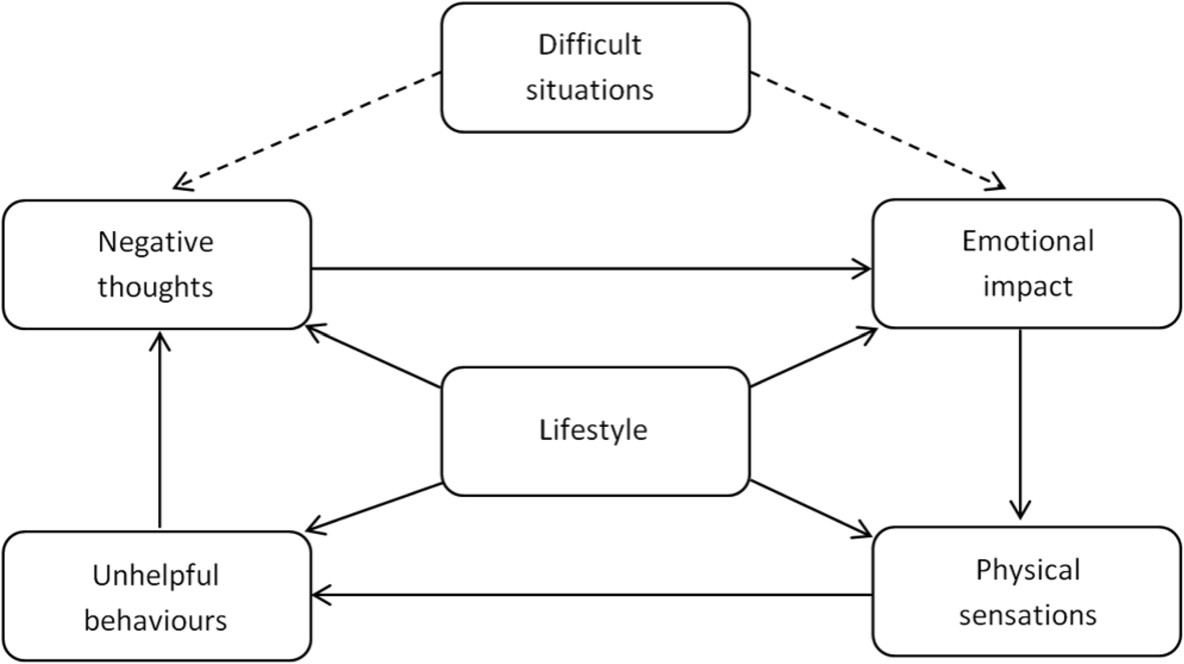
The Lifestyle Balance Model

When an individual first uses BFO, they complete a psychometric assessment developed by the authors, the ‘Recovery Progression Measure’ (RPM: Elison et al. 2016a; 2017b, which is contained within the program. The RPM measures baseline levels of functioning, and treatment-related changes in functioning, across six domains; ‘negative thoughts’, ‘emotional impact’, ‘unhelpful behaviors’, ‘difficult situations’, ‘physical sensations’, and ‘lifestyle’. Data generated via completion of the RPM is then utilized by the BFO program to populate a visual depiction of a six-domain biopsychosocial model, the ‘Lifestyle Balance Model’ (LBM: Davies et al. 2015). The LBM forms the theoretical underpinnings of the program and is based on the five-factor model used in cognitive behavioral therapy (Greenberger and Padesky 1995; Williams and Chellingsworth 2010). The LBM (see Fig. 1) acts as a clinical formulation to help the user understand the domains of their functioning that may be implicated in their substance misuse.

Based on RPM scores, each of the six domains of the visual depiction of the LBM are colored either green, amber or red. This ‘traffic light’ system indicate respectively, ‘little’, ‘moderate’ or ‘significant’ impairment in each of the six domains. Tailored advice then guides the user to concentrate on completing intervention strategies contained in the program that are aligned to domains of functioning in the LBM where they may be experiencing the greatest levels of impairment (amber and red domains of the LBM). However, users are also encouraged to complete and interventions within green areas to help build long-term resilience.

Table 1 provides an overview of the clinical content of BFO and the theoretical underpinnings of individual intervention strategies within the program. Table 1 maps the clinical content of BFO onto individual behavior change techniques (BCTs) from the BCT taxonomy (V1) (Dugdale et al. 2016b). The BCT taxonomy (V1) provides a standardized means of describing the clinical content of complex behavioral change interventions (Michie et al. 2011).

**Table 1:** Breaking Free Online content

Strategies included in Breaking Free Online	Description of strategy	Therapeutic approaches underpinning strategies	BCT taxonomy (V1) techniques (number in taxonomy)
Lifestyle balance model (individualized diagram)	Generic formulation; Idiosyncratic formulation; Personalized feedback; Case formulation – understand the links between situations, thoughts, emotions, behaviors, physical sensations and lifestyle	Node-link mapping (International Treatment Effectiveness Program (ITEP); Cognitive-behavioral therapy (CBT)	Information about antecedents (4.2); Information about health consequences (5.1); Salience of consequences (5.2); Information about social and environmental consequences (5.3); Information about emotional consequences (5.6)
Understanding your difficult situations	Assessment; Self-monitoring; Standardized measures; Psycho-education on impact of problematic situations; Intervention to help people in distress access support	All structured therapeutic approaches; Psycho-education; Guided self-help	Social support (unspecified) (3.1); Reduce negative emotions (11.2)
Managing your risky situations: Recognize– avoid–cope	Recognize–avoid–cope; Relapse prevention for coping with environmental/situational/emotional triggers; Creating action plans on how to avoid or cope in high risk situations	Relapse prevention; Refusal skills	Problem solving (1.2); Action planning (1.4); Instruction on how to perform a behavior (4.1); Behavioral practice/rehearsal (8.1); Behaviour substitution (8.2); Avoidance/reducing exposure to cues for the behavior (12.3); Goal setting (behavior) (1.1); Problem solving (1.2); Action planning (1.4)
Understanding your negative thoughts	Psycho-education on impact on negative thoughts	Psycho-education; Guided self-help	Information about antecedents (4.2); Information about health consequences (5.1); Salience of consequences (5.2); Information about social and environmental consequences (5.3); Information about emotional consequences (5.6)
Escaping your mind traps	Mind traps; Cognitive restructuring; Challenge thoughts that may be unhelpful	International Treatment Effectiveness Program (ITEP); Cognitive-behavioral therapy (CBT)	Re-attribution (4.3); Framing-reframing (13.2)
Understanding your emotions	Psycho-education on impact on emotions	Psycho-education; Guided self-help	Information about antecedents (4.2); Information about health consequences (5.1); Salience of consequences (5.2); Information about social and environmental consequences (5.3); Information about emotional consequences (5.6)
Shifting your focus	Attention narrowing; Attention switching; Emotional regulation; Recognize/understand/normalize emotions; Developing more appropriate coping strategies	Coping strategy enhancement (CSE); Mindfulness-based cognitive therapy	Information about emotional consequences (5.6); Behavioral practice/rehearsal (8.1); Reduce negative emotions (11.2); Problem solving (1.2); Social support (unspecified) (3.1); Behavioral practice/rehearsal (8.1); Distraction (12.4)
Understanding your physical sensations	Psycho-education on impact of physical sensations	Psycho-education; Guided self-help	Information about antecedents (4.2); Information about health consequences (5.1); Salience of consequences (5.2); Information about social and environmental consequences (5.3); Information about emotional consequences (5.6)
Surfing your cravings and urges	Urge surfing; Body scanning; Relapse prevention-based techniques	Mindfulness-based cognitive therapy	Instruction on how to perform a behavior (4.1); Behavioral practice/rehearsal (8.1); Reduce negative emotions (11.2)
Understanding your unhelpful behaviors	Psycho-education on impact of destructive behaviors	Psycho-education; Guided self-help	Information about antecedents (4.2); Information about health consequences (5.1); Salience of consequences (5.2); Information about social and environmental consequences (5.3); Information about emotional consequences (5.6)
Planning your time positively	Activity scheduling; Behavioral activation; Encourage new behaviors via positive feedback; Increase activity to increase energy levels and relieve boredom	Cognitive-behavioral therapy (CBT)	Non-specific reward (10.3); Non-specific incentive (10.6); Reward approximation (14.4); Rewarding completion (14.5); Goal setting (behavior) (1.1); Action planning (1.4)
Understanding your lifestyle	Psycho-education on impact of lifestyle; Creating SMART goals for recovery	Psycho-education; Guided self-help	Goal setting (behavior) (1.1); Problem solving (1.2); Goal setting (outcome) (1.3); Action planning (1.4)
Achieving your life goals	Goal-setting Increase treatment engagement and retention. Increase readiness to change behavior	Motivational enhancement therapy (MET); Implementation intentions	Non-specific reward (10.3); Focus on past success (15.3); Action planning (1.4)
Progress check	Monitor behavior to provide feedback about progress towards goals; Encourage new behaviors via positive feedback	Self-monitoring	Self-monitoring of behavior (2.3); Feedback on outcome(s) of behavior (2.7)
Information on alcohol and drugs	Psycho-education on effects of alcohol and drugs; Reduce negative or fatal consequences of substance using behaviors	Harm reduction; Psycho-education	Information about health consequences (5.1); Salience of consequences (5.2); Behaviour substitution (8.2); Habit reversal (8.4)

The BFO program has been designed to be used by individuals as either a stand-alone or adjunct treatment program alongside standard treatment, and as either self-help or as CAT with support by practitioners, keyworkers, peer-mentors or other supporters. However, in this study BFO will be delivered as a supplement to standard treatment. Consultation with HMPPS has ensured that all intervention strategies in the program are appropriate for the prison setting and comply with HMPPS quality assurance, security and information assurance processes. For this study, the BFO program will be comprised of 8 sessions which will be run over 4-weeks, with two sessions held each week.

### Standard treatment

Both study groups will receive standard treatment as part of the study design. It is expected that there will be a degree of heterogeneity within both the BFO and control groups in terms of the ‘standard treatment’ each participant receives. Information regarding the specific standard treatments each participant receives will be collected, including the specific standard treatments each participant receives, the number of sessions completed, what kind of practitioner has delivered it, and also the medications each participant may have been prescribed during the course of the study (see Additional file 1: Appendix B). These data will allow comparisons to be made between different control treatments.

In terms of the psychosocial and behavioral support available in the participating prison, low-intensity group-based interventions are usually delivered by key-workers in alcohol and drug misuse services and include techniques such as motivational interviewing and contingency management. These group-based interventions will be delivered with groups of participants that are of a similar size to the group sessions of BFO, i.e. groups of 10 participants. In order to avoid violation of the stable unit treatment value assumption, participants in the BFO group will receive their group-based standard treatment in groups that are run separately from the group-based standard treatments the control group receive.

More formal psychological therapies are usually delivered by specialist psychological therapists through CBT based interventions and are delivered on a one-to-one basis. All standard treatment sessions have a duration range of 30–60 min and take place once or twice a week for a period of approximately 4–12 weeks. The number of interventions each participant will receive may vary. During treatment concomitant alcohol and drug/s use may be permitted, as well as any prescribed medication (detoxification included).

### Procedure

Site investigators from the research team will work with the support of members of the team of substance misuse practitioners working in the prison to inform potential participants of the study. Site investigators will be responsible for completing screening, consent and randomization. Site investigators and practitioners will be responsible for conducting baseline and 4-week post-baseline assessments. Practitioners will be responsible for delivering both BFO and the standard treatments received by the two study groups. Site investigators will be responsible for conducting 3- and 6-month follow-up assessments. All site investigators and practitioners have been vetted and security cleared to work in prisons by HMPPS and have enhanced clearance via the UK Government Disclosure Barring Service. The site investigators have trained all participating practitioners in delivering BFO as CAT and have also conducted training around RCT methodology. All practitioners are trained and experienced facilitators of structured substance misuse interventions. Both site investigators and practitioners have received training in ethics and confidentiality issues when working with offenders in secure settings.

All prospective participants that potentially meet the study criteria will be informed of the study’s objectives and requirements using the Participant Information Sheet and Informed Consent Form before any screening procedures are performed. If willing to participate in the study, participants will be requested to provide written consent after being given sufficient time to consider their participation and having had the opportunity to ask for further details. During the informed consent procedure, participants will be provided with information about the fact they can withdrawal from the study at any time without giving a reason, and without their withdrawal affecting their recovery and rehabilitation support. Additionally, they will be assured that when their data are collected, no identifiable information can be traced back to them, and that their data will be fully anonymized.

The Informed Consent Form will be signed and dated by both the participant and the site investigator, and the participant’s preferred mode of contact (e.g. telephone, email etc.) and contact details for the completion of follow-up assessments will also be noted at this point. The participant will be provided with a copy of the signed consent form and the Participant Information Sheet. The original consent forms will be retained in a secure storage facility separately from source data to protect against breach of privacy and participant anonymity.

If written consent is given, each participant will then be screened for eligibility by the site investigator before then being randomized to either the BFO or control group. Randomization will occur at the level of the individual participants, with participants being assigned to one of the study groups following the generation of a random allocation sequence via the *Research Randomizer* (from the Social Psychology Network- Urbaniak and Plous 2011). Sequentially-numbered opaque sealed envelopes containing the treatment group that the participant will be allocated to will be delivered to the research site prior to commencement of the study. The participant number will be determined according to the order of enrolment in the study. The site investigator will assign and open one sealed envelope per participant. A screening log, including the participant number and treatment group assigned by randomization and any subsequent reason/s for exclusion from the study (if applicable), will then be completed by the site investigator.

No longer than 2-weeks after being randomized and completing the eligibility screening, both study groups will complete a battery of assessments to collect data around primary and secondary outcomes and demographic information including age and ethnicity. This battery of assessment will be delivered digitally via desktop computers within the prison IT suites. The assessment for the BFO group will be completed within the BFO program. The control group will complete the measures via a specially developed digital assessment platform which will deliver the same assessment that is included in the BFO program, but without providing access to any of the digital intervention content provided by BFO.

Study groups will then complete a period of 4-weeks of substance misuse treatment, of either i) BFO plus standard treatment, or ii) standard treatment only. For the BFO group, the 4-week intervention will be delivered to groups of approximately 10 participants at a time, so it is estimated that approximately 6–8 groups will need to run successively to achieve the sample size required, in order to account for some attrition between baseline and 4-week post-treatment assessments. BFO group participants will receive two sessions of BFO each week, alongside any standard treatment they may be engaging with.

Online access to the BFO program is granted via the activation of an access code given to the participant at the alcohol and drug misuse service by authorized practitioners. To activate the access card and create a personal account, the participant must enter a username and password of his choice along with the access code. The practitioners will be able to assist with the online access if required. The participants must also agree to the Terms & Conditions of using BFO, which are in accordance with the Participant Information Sheet and Informed Consent Form and conform to the European Union General Data Protection Regulation around the use of digitally captured personal data. The practitioners must ensure that they log out of BFO at the end of each treatment session to protect the confidentiality of the data.

Procedures to enhance retention of participants during the 4-week treatment period will include practitioners providing ongoing support during weekly key-working sessions, which all offenders in the prison will routinely receive during the standard substance misuse treatment. When participants do dropout of the study, new participants will be recruited and randomized to replace dropouts, in order to ensure the required sample size of 120 participants (60 per group) complete the 4-week treatment period and provide post-treatment data. However, in line with ITT principles, all randomized participants, including dropouts, will be included in the final analyses (see ‘Data Analysis’ section). After completion of the 4-week treatment period, participants in both groups will complete the same battery of digital psychometric assessments on the prison desktop computers.

Follow-up assessments will then also be completed at 3- and 6-months post-treatment with all participants. Follow-up assessments may be completed either in the community if offenders have been released, or in prison if they are still serving their sentence. The site investigators will work with the practitioners delivering both BFO and standard treatments to determine which participants have been released back to the community since completing their 4-week treatment period, and which participants are still serving their sentence in the participating prison. Additionally, those participants who have been transferred to a different prison will also be identified.

Depending on participants’ preference, the site investigators will contact all participants who have been released back to the community via telephone or email, and their assessment will be completed either over the phone, or via a link to an online version of the assessment. The preferred contact details for each participant that were collected at the time of consent and randomization will be used. For those participants who are still serving a prison sentence, or have been reconvicted and are serving a new sentence, or have been transferred to a different prison, they will be visited by the site investigator at their current prison and their follow-up assessment will be completed there.

By following the procedure described above, each participant will take part in the study for a total of approximately 7-months, including 4-weeks of treatment and 3- and 6-month follow-up assessments. It is likely that the study will run for a total of approximately 18-months, which will include enough time for recruitment, running enough successive 4-week long BFO groups to achieve the required sample size, and completion of all 3- and 6-month follow-up assessments.

### Measures

The primary outcome will be self-reported substance use which will be calculated using answers to two questions i) ‘on a typical day, how many (unit of measurement) of (substance) are you using?’, and ii) ‘in a typical week, how many days are you using (substance)?’. Given the research setting, i.e. a Category D ‘open prison’ where offenders spend time in the community each week, it is more likely that any substance use outcomes will be due to genuine treatment effects, than they would be in a more highly secure prison setting with fewer opportunities to access and use substances. Information about weekly substance use before each participants’ current prison sentence will also be collected.

A number of secondary outcomes will also be measured and will come from standardized psychometric assessments of biopsychosocial functioning, which will include:

i) Severity of substance dependence: This will be measured using the Severity of Dependence Scale (SDS; Gossop et al. 1995), which is a 5-item, 4-point Likert scale measuring psychological dependence on illicit drugs, that has been previously used in studies of persistent drug use in prison populations (Strang et al. 2006) and studies of programs to address substance related offending behaviors (Crane and Blud 2012). It has been demonstrated to have excellent reliability with an alpha coefficient of .89.
ii) Mental health sequelae: This will be measured using the Patient Health Questionnaire (PHQ-4; Kroenke et al. 2009), which is a 4-item, 4-point Likert scale measuring depression and anxiety, which has been demonstrated to have excellent internal reliability (alpha = .81), with scores on the PHQ-4 having been demonstrated to converge with scores on other measures of anxiety and the 20-Item Short Form Health Survey.
iii) Quality of life: This will be measured using 5 items from the World Health Organization Quality of life measure (WHOQoL-BREF; Skevington et al. 2004): A total of 5 items (items 1, 2, 17, 18, 20) from the WHOQoL-BREF have been selected for measuring general quality of life. Taken as a whole measure, the WHOQoL-BREF is a 26-item, 5-point Likert scale containing items measuring 4 main domains - physical, psychological, social and environmental life satisfaction. As only the first 5 items of the WHOQoL-BREF are being used, reliability and validity analyses will be conducted on data generated from these 5 items. Internal reliability which will be examined using Cronbach’s alpha, and concurrent validity will be examined by correlating scores on the 5 WHOQoL-BREF items against scores on the other measures included in the study measuring constructs related to quality of life.
iv) Biopsychosocial functioning: This will be measured using the Recovery Progression Measure (RPM; Elison et al. 2016a, b; Elison et al. 2017a, b, c, d), which is a 36-item measure comprising 6 ‘impact slider’, 11-point Likert scale items each measuring level of severity of impairment in the following 6 domains of functioning; difficult situations, negative thoughts, emotions, unhelpful behaviors, physical sensations, lifestyle. In addition, the RPM contains 30 dichotomous ‘yes/no’ response items measuring presence or absence of specific biopsychosocial issues within each of the 6 domains. Statistical standardization analyses based on a sample of 2218 service users seeking support for substance misuse found the overall RPM scale to have excellent reliability with an alpha coefficient of .89. The RPM has also been found to be a valid measure with scores from 9208 service users converging significantly with those on standardized psychometric measures of mental health and substance dependence (*p* < .0001).

In addition to the standardized measures described above, socio-demographic data will also be collected via the digital assessment completed by both study groups, including age and ethnicity. Additional socio-demographic data will be collected via paper/pen assessment when participants are first randomized, including educational level achieved, employment status before entering prison, and marital status (See Additional file 1: Appendix A), and what standard treatments each participant has received (see Additional file 1: Appendix B). This will allow comparisons of the two study groups to be made, and also comparisons of all participants with data published each year by the UK Ministry of Justice describing the broader prison population in England and Wales.

When follow-up assessments are conducted at 3- and 6-months, each participant will also be asked about any further involvement with criminal justice authorities since being released from prison, if they have been released from prison in the interim, and if so, the nature of this involvement with the authorities, e.g. being arrested, any court appearances etc. (See Additional file 1: Appendix C). For ethical and legal reasons, these questions will be restricted to questions that enquire as to criminal justice system involvement only, as opposed to asking participants about any crimes they may have committed that may not have been discovered by the authorities.

### Data analysis

Quantitative data will be analyzed and reported using SPSS® Version 25.0 (or later) with all analyses to be performed as per the statistical analysis plan. The appropriate 95% - confidence interval will be applied. The *all-randomized population* will consist of all participants in the study that have been screened and allocated to either the i) BFO plus standard treatment, or, ii) standard treatment only group. The *per-protocol population* will consist of all participants randomized into the study who have completed the 4-week treatment period as well as all follow-up assessments.

Analyses will be performed on the basis of an ITT population, with the all-randomized population included in the analyses. No participants will be excluded from the ITT analyses, i.e. those who have withdrawn, been lost to follow-up, or have provided incomplete outcomes data. Separate analyses will also be conducted on the per-protocol population who have provided at least one set of follow-up data. Outcomes from these two analyses (ITT, per-protocol population) will be compared to examine whether missing data may have had an impact on reliability of conclusions formed around comparative effectiveness of the two study conditions.

Previous analyses (e.g. Elison et al. 2015a, b, c) indicate that data will likely be non-normally distributed in which case nonparametric repeated-measures analyses of covariance (ANCOVA) using appropriate distribution such as Poisson distribution, will be used to compare the study groups at 4-weeks post-treatment and 3- and 6-months follow-up on self-reported substance use, substance dependence, mental health sequelae, biopsychosocial functioning and quality of life. However, normality will be tested when data are available for analyses, and appropriate distributions will be applied.

Specialist statistical support has been sought from colleagues at one of the collaborating academic institutions (University of Manchester), who have provided advice during protocol development and will provide ongoing advice throughout the study. When analyzing differences between the two study groups at each of the outcomes data time-points, (4-weeks post-treatment, 3- and 6-month follow-ups) baseline scores will be controlled for as post-treatment between-group differences may reflect both treatment effects and also group differences at baseline that randomization may not have addressed. Differences between the groups in post-treatment scores will be ascertained using estimated marginal means.

Effect sizes will also be calculated to examine robustness of between-group differences and within-group changes over time, using partial eta squared (ὴ^2^), which is an appropriate measure of effect size for ANCOVA. The numbers of participants fulfilling clinical threshold scores for substance dependence, depression and anxiety at baseline and post-treatment will also be examined.

### Power calculation

Since the study is a parallel-group comparison, equal numbers of participants will be required for each of the groups; i) BFO plus standard treatment, and, ii) standard treatment only. The study projection of the sample size will require 60 evaluable participants in each treatment group to achieve enough power (assuming power of 0.80 with α = .05) with an allowance of 50% attrition at 3- and 6-months follow-up, which is in line with previous studies with offender populations receiving interventions for substance misuse in correctional settings (e.g. Crisanti et al. 2014). In addition, this level of attrition is also seen in substance misuse intervention research more generally (e.g. Brorson et al. 2013) and in many digital interventions studies (e.g. Eysenbach 2005). To obtain a total of 120 evaluable participants, it is estimated 240 participants will need to be recruited and screened.

These estimations have been based on previous samples used for assessments of CAT (Carroll et al. 2008), some of which have used longitudinal statistical analyses (Koski-Jännes et al. 2009; Kypri et al. 2008). It is envisaged that the estimated evaluable participant population will be sufficiently large to enable meaningful descriptive comparisons to be performed.

## Discussion

This protocol describes the methodology for an RCT to examine the efficacy of a CAT program for substance-involved offenders, Breaking Free Online (BFO) Health and Justice, when delivered alongside standard treatment, compared with standard treatment only, in a prison setting. This program is the first digital offender management program to be accredited and commissioned by the UK Ministry of Justice, and to date, this is the first RCT of a digital treatment program for offenders to be conducted within the UK prison estate.

Published research examining effectiveness of both the community treatment setting version of the program Elison et al. (2015a, b) and the criminal justice setting version described in this protocol (Davies et al. 2017; Elison et al. 2015c) has suggested that the program may be effective in supporting substance misusing individuals to significantly reduce their substance use and dependence. In addition, the program may significantly reduce the severity of mental health difficulties and biopsychosocial impairment and improve quality of life (Elison et al. 2014a; Elison et al. 2015a, b; Elison et al. 2017d).

Other published research has examined mechanisms of action of BFO in a sample of participants engaging with the program in community-based treatment settings (Elison et al. 2017c) which has demonstrated the primacy of cognitive change to instigating behavioral change. Completion of cognitive restructuring strategies in the program has been demonstrated to be associated with multiple aspects of behavioral and biopsychosocial improvements. In addition, this research has demonstrated that individuals using BFO follow tailoring advice provided by the program, which suggests that users spend more time working on intervention strategies associated with their domains of most significant biopsychosocial impairment. Therefore, for the group in this RCT who will be engaging with BFO alongside standard treatment, mechanisms of action analyses will be replicated, to examine the mechanisms of action of the program when implemented in a criminal justice setting.

Potential limitations of the methodology include the fact that it will be difficult for the investigators or the practitioners working in the prison substance misuse service to be blinded to the allocation of participants to each of the two study groups. This is because the investigators will have to randomize participants and then organize the BFO groups in the prison, and so they will need to know which group each participant is randomized to in order to do this. Although the practitioners will not immediately know which group each participant has been allocated to, very shortly after randomization they will receive a list of all participants in the BFO group. This is because they will need to check attendance to the group, which is particularly important in a secure prison environment in which the whereabouts of individual offenders must be accounted for at all times. It is also a requirement of prison regimes in the UK for any member of staff facilitating a group session to know ahead of the session which offenders will be attending. This is so staff can be aware ahead of the session of any special circumstances surrounding each offender that may need to be taken into account or may pose a risk for any reason, for example if a specific offender had recently had any emotional or behavioral difficulties etc.

Another limitation lies in the fact that the level of randomization is at the level of individual offenders, which means that intervention group participants and control group participants will have the opportunity to interact with one another within the prison. This may result in members of the intervention group discussing content of BFO with control group participants, which may contaminate the outcomes and violate the stable unit treatment value assumption. However, this study has to be conducted in a very specific kind of prison environment, which limited the number of potential research sites and made the option of randomization at the level of the research site impractical. Indeed, recruiting prisons to participate in research is difficult generally, given the unique challenges faced within such secure environments and the additional challenges posed by issues such as understaffing in most UK prisons – this makes it difficult for prisons to participate in additional activities outside of the core prison regime. And from a methodological perspective, the study has to be conducted in an open prison where participants may conceivably have an opportunity to continue to use substances, although the authors note there is still significant illicit substance use in the UK and other countries, even in high-secure prisons.

There may also be a limitation inherent due to the choice to include an open prison in the study. Open prisons generally contain low-risk offenders who may have already demonstrated that they have made good progress in their rehabilitation. Therefore, a ‘floor effect’ may occur that could artificially under-estimate the effectiveness of the BFO program if participants largely start at a baseline level of relatively good biopsychosocial functioning. Finally, a significant limitation may lie with the possible attrition rates that may be expected from a study that includes substance-involved offenders as participants, as it can be particularly difficult to maintain engagement with individuals who may have issues around substance misuse and offending. Although measures can be put in place to enhance retention of participants whilst they are still serving their prison sentence, such as weekly key-working sessions with practitioners as part of standard treatment, it may be particularly challenging to retain participants in the study once they are released back to the community. Every attempt will be made to contact participants and obtain follow-up data, and it is hoped that as most participants will be in contact with probation services, this may provide a means of contacting participants as they complete their sentences in the community.

## Additional file

Additional file 1: Appendix A-C. (DOC 36 kb)

## Abbreviations

ANCOVA: Analysis of covariance
CAT: Computer-assisted therapy
CBT: Cognitive-behavioural therapy
HMPPS: Her Majesty’s Prison and Probation Service
ITT: Intention to treat
LBM: Lifestyle Balance Model
NICE: National Institute of Health and Care Excellence
PHQ: Patient Health Questionnaire
RCT: Randomised controlled trial
RPM: Recovery Progression Measure
SDS: Severity of dependence scale
VC: Virtual campus
WHOQoL-BREF: World Health Organisation Quality of Life Scale

## References

[CR1] American Psychiatric Association. (2000). *Diagnostic and statistical manual of mental disorders- text revision (DSM-IV-TR).* Virginia: American Psychiatric Association.

[CR2] Ball JC, Shaffer JW, Nurco DN (1983). The day to-day criminality of heroin addicts in Baltimore—A study in the continuity of offence rates. Drug and Alcohol Dependence.

[CR3] Beck Aaron T. (1993). Cognitive therapy: Past, present, and future. Journal of Consulting and Clinical Psychology.

[CR4] Beck, A. T., Wright, F. D., Newman, C. F., & Liese, B. S. (2011). *Cognitive therapy of substance abuse*. New York: Guilford Press. 8289917

[CR5] Bennett T, Holloway K (2009). The causal connection between drug misuse and crime. British Journal of Criminology.

[CR6] Bennett T, Holloway K, Farrington D (2008). The statistical association between drug misuse and crime: A meta-analysis. Aggression and Violent Behavior.

[CR7] Best D, Sidwell C, Gossop M, Harris J, Strang J (2001). Crime and expenditure amongst Polydrug misusers seeking treatment the connection between prescribed methadone and crack use, and criminal involvement. British Journal of Criminology.

[CR8] Bickel WK, Marsch LA, Buchhalter AR, Badger GJ (2008). Computerized behavior therapy for opioid-dependent outpatients: A randomized controlled trial. Experimental and Clinical Psychopharmacology.

[CR9] Boden JM, Fergusson DM, Horwood LJ (2012). Alcohol misuse and violent behavior: Findings from a 30-year longitudinal study. Drug & Alcohol Dependence.

[CR10] Brorson Hanne H., Ajo Arnevik Espen, Rand-Hendriksen Kim, Duckert Fanny (2013). Drop-out from addiction treatment: A systematic review of risk factors. Clinical Psychology Review.

[CR11] Budd T, Collier P, Mhlanga B, Sharp C, Weir G (2005). Levels of self-report offending and drug use among offenders: Findings from the Criminality Surveys.

[CR12] Carroll K, Ball S, Martino S, Nich C, Babuscio T, Nuro K, Rounsaville B (2008). Computer-assisted delivery of cognitive-behavioral therapy for addiction: A randomized trial of CBT4CBT. American Journal of Psychiatry.

[CR13] Comiskey CM, Stapleton R, Kelly PA (2012). Ongoing cocaine and benzodiazepine use: Effects on acquisitive crime committal rates amongst opiate users in treatment. Drugs: Education, Prevention and Policy.

[CR14] Craig P, Dieppe P, Macintyre S, Michie S, Nazareth I, Petticrew M (2008). Developing and evaluating complex interventions: The new Medical Research Council guidance. British Medical Journal.

[CR15] Crane MAJ, Blud L (2012). The effectiveness of prisoners addressing substance related offending (P-ASRO) programme: Evaluating the pre and post treatment psychometric outcomes in an adult male category C prison. British Journal of Forensic Practice.

[CR16] Crisanti AS, Case BF, Isakson BL, Steadman HJ (2014). Understanding study attrition in the evaluation of jail diversion programs for persons with serious mental illness or co-occurring substance use disorders. Criminal Justice and Behavior.

[CR17] Davies, G., Elison, S., Ward, J., Laudet, A. (2015). The role of lifestyle in perpetuating substance dependence: A new explanatory model, the Lifestyle Balance Model. *Substance Abuse, Treatment, Prevention and Policy, 10*(2), e1–18. 10.1186/1747-597X-10-2 PMC4326198 25595205

[CR18] Davies, G., Ward, J., Elison, S., Weston, S., Dugdale, S., & Weekes, J. (2017). Implementation and evaluation of the 'Breaking Free Online' and 'Pillars of Recovery' treatment and recovery programmes for substance-involved offenders: Reflections from the North-West prisons ‘Gateways’ pathfinder. *Advancing Corrections*, *3,* 95–113.

[CR19] Dugdale Stephanie, Elison Sarah, Davies Glyn, Ward Jonathan, Dalton Martha (2016). A Qualitative Study Investigating the Continued Adoption of Breaking Free Online Across a National Substance Misuse Organisation: Theoretical Conceptualisation of Staff Perceptions. The Journal of Behavioral Health Services & Research.

[CR20] Dugdale S, Elison S, Ward J, Davies G, Dalton M (2016). Using the Transtheoretical model to explore the impact of peer mentoring on peer mentors’ own recovery from substance misuse. Journal of Groups in Addiction and Recovery.

[CR21] Dugdale, S., Ward, J., Hernen, J., Elison, S., Davies, G., & Donkor, D. (2016b). Using the behavior change technique taxonomy v1 to conceptualize the clinical content of Breaking Free Online: A computer-assisted therapy program for substance use disorders. *Substance Abuse Treatment, Prevention, and Policy*, *11*(1), 26. 10.1186/s13011-016-0069-y PMC4957914 27449786

[CR22] Elison Sarah, Davies Glyn, Ward Jonathan (2015). Effectiveness of Computer-Assisted Therapy for Substance Dependence Using Breaking Free Online: Subgroup Analyses of a Heterogeneous Sample of Service Users. JMIR Mental Health.

[CR23] Elison Sarah, Davies Glyn, Ward Jonathan (2015). An Outcomes Evaluation of Computerized Treatment for Problem Drinking using Breaking Free Online. Alcoholism Treatment Quarterly.

[CR24] Elison, S., Davies, G., & Ward, J. (2016a). Initial development and psychometric properties of a new measure of substance misuse ‘recovery progression’: The Recovery Progression Measure (RPM). *Substance Use and Misuse, 51*(9), 1195–1206. 10.3109/10826084.2016.1161052 27191336

[CR25] Elison S, Davies G, Ward J, Weston S, Dugdale S, Weekes J (2017). Using the “recovery” and “rehabilitation” paradigms to support desistance of substance-involved offenders: Exploration of dual and multi-focus interventions. Journal of Criminological Research, Policy and Practice.

[CR26] Elison S, Dugdale S, Ward J, Davies G (2017). The ‘rapid recovery progression measure’ (rapid-RPM) a brief assessment of psychosocial functioning change during problematic substance use recovery progression. Substance Use and Misuse.

[CR27] Elison Sarah, Jones Andrew, Ward Jonathan, Davies Glyn, Dugdale Stephanie (2017). Examining effectiveness of tailorable computer-assisted therapy programmes for substance misuse: Programme usage and clinical outcomes data from Breaking Free Online. Addictive Behaviors.

[CR28] Elison Sarah, Ward Jonathan, Davies Glyn, Lidbetter Nicky, Hulme Daniel, Dagley Mike (2014). An outcomes study of eTherapy for dual diagnosis using Breaking Free Online. Advances in Dual Diagnosis.

[CR29] Elison Sarah, Ward Jonathan, Davies Glyn, Moody Mark (2014). Implementation of computer-assisted therapy for substance misuse: a qualitative study of Breaking Free Online using Roger's diffusion of innovation theory. Drugs and Alcohol Today.

[CR30] Elison Sarah, Ward Jonathan, Williams Chris, Espie Colin, Davies Glyn, Dugdale Stephanie, Ragan Kathryn, Chisnall Leanne, Lidbetter Nicky, Smith Keith (2017). Feasibility of a UK community-based, eTherapy mental health service in Greater Manchester: repeated-measures and between-groups study of ‘Living Life to the Full Interactive’, ‘Sleepio’ and ‘Breaking Free Online’ at ‘Self Help Services’. BMJ Open.

[CR31] Elison Sarah, Weston Samantha, Davies Glyn, Dugdale Stephanie, Ward Jonathan (2015). Findings from mixed-methods feasibility and effectiveness evaluations of the “Breaking Free Online” treatment and recovery programme for substance misuse in prisons. Drugs: Education, Prevention and Policy.

[CR32] Elison Sarah, Weston Samantha, Dugdale Stephanie, Ward Jonathan, Davies Glyn (2016). A Qualitative Exploration of U.K. Prisoners’ Experiences of Substance Misuse and Mental Health Difficulties, and the Breaking Free Health and Justice Interventions. Journal of Drug Issues.

[CR33] Eysenbach Gunther (2005). The Law of Attrition. Journal of Medical Internet Research.

[CR34] Fazel S, Yoon IA, Hayes AJ (2017). Substance use disorders in prisoners: An updated systematic review and meta-regression analysis in recently incarcerated men and women. Addiction.

[CR35] Goldstein Paul J. (1985). The Drugs/Violence Nexus: A Tripartite Conceptual Framework. Journal of Drug Issues.

[CR36] Gossop M, Darke S, Griffiths P, Hando J, Powis B, Hall W, Strang J (1995). The severity of dependence scale (SDS): Psychometric properties of the SDS in English and Australian samples of heroin, cocaine and amphetamine users. Addiction.

[CR37] Gossop M, Marsden J, Stewart D, Rolfe A (2000). Reductions in acquisitive crime and drug use after treatment of addiction problems: 1-year follow-up outcomes. Drug and Alcohol Dependence.

[CR38] Greenberger, D., & Padesky, C. A. (1995). *Mind over mood: Change how you feel by changing the way you think*. New York: Guilford Press.

[CR39] Hayhurst KP, Jones A, Millar T, Pierce M, Davies L, Weston S, Donmall M (2013). Drug spend and acquisitive offending by substance misusers. Drug & Alcohol Dependence.

[CR40] Home Office (2016). Drug misuse: Findings from the 2015/16 crime survey for England and Wales: Second edition.

[CR41] Hough M (2002). Drug user treatment within a criminal justice context. Substance Use & Misuse.

[CR42] Inciardi JA (1979). Heroin use and street crime. Crime & Delinquency.

[CR43] Jones A, Weston S, Moody A, Millar T, Dollin L, Anderson T, Donmall M (2007). The drug treatment outcomes research study (DTORS): Baseline report.

[CR44] Koski-Jännes A, Cunningham J, Tolonen K (2009). Self-assessment of drinking on the internet—3-, 6-and 12-month follow-ups. Alcohol and Alcoholism.

[CR45] Kroenke K, Spitzer RL, Williams JB, Löwe B (2009). An ultra-brief screening scale for anxiety and depression: The PHQ–4. Psychosomatics.

[CR46] Kypri K, Langley JD, Saunders JB, Cashell-Smith ML, Herbison P (2008). Randomized controlled trial of web-based alcohol screening and brief intervention in primary care. Archives of Internal Medicine.

[CR47] Lundholm L, Haggård U, Möller J, Hallqvist J, Thiblin I (2013). The triggering effect of alcohol and illicit drugs on violent crime in a remand prison population: A case crossover study. Drug & Alcohol Dependence.

[CR48] Marlatt, G., Bowen, S., Chawla, N., & Witkiewitz, K. (2010). Mindfulness-based relapse prevention for substance abusers: Therapist training and therapeutic relationships. In Z. Segal, S. Hick, & T. Bien (Eds.), *Mindfulness and the therapeutic relationship*. New York: Guilford Press.

[CR49] Marlatt, G. A., & Donovan, D. M. (2005). *Relapse prevention: Maintenance strategies in the treatment of addictive behaviors*. New York: Guilford Press.

[CR50] McGlothlin WH, Anglin MD, Wilson BD (1978). Narcotic addiction and crime. Criminology.

[CR51] Michie S, Hyder N, Walia A, West R (2011). Development of a taxonomy of behaviour change techniques used in individual behavioural support for smoking cessation. Addictive Behaviors.

[CR52] Moore BA, Fazzino T, Garnet B, Cutter CJ, Barry DT (2011). Computer-based interventions for drug use disorders: A systematic review. Journal of Substance Abuse Treatment.

[CR53] Moore G. F., Audrey S., Barker M., Bond L., Bonell C., Hardeman W., Moore L., O'Cathain A., Tinati T., Wight D., Baird J. (2015). Process evaluation of complex interventions: Medical Research Council guidance. BMJ.

[CR54] National Treatment Agency for Substance Misuse. (2006a). *Models of care for treatment of adult drug misusers: Update 2006*. London: NHS.

[CR55] National Treatment Agency for Substance Misuse. (2006b). *Review of the effectiveness of treatment for alcohol problems*. London: NHS.

[CR56] NICE. (2007). *Drug misuse in over 16s: Psychosocial interventions: Clinical guideline [CG51]*. London: National Institute for Health and Care Excellence. 31877006

[CR57] NICE. (2011). *Alcohol-use disorders: diagnosis, assessment and management of harmful drinking and alcohol dependence: Clinical guideline [CG115]*. London: National Institute for Health and Care Excellence. 39808037

[CR58] NICE. (2012). *Drug use disorders in adults: Quality standard [QS23]*. London: National Institute for Health and Care Excellence.

[CR59] PHE. (2016). *The Public Health Burden of Alcohol and the Effectiveness and Cost-Effectiveness of Alcohol Control Policies: An evidence review*. London: Public Health England.

[CR60] PHE. (2017). *Adult substance misuse statistics from the National Drug Treatment Monitoring System (NDTMS) - 1 April 2016 to 31 March 2017*. London: Public Health England.

[CR61] Phillips P. (2000). Substance misuse, offending and mental illness: a review. Journal of Psychiatric and Mental Health Nursing.

[CR62] Schroeder RD, Giordano PC, Cernkovich SA (2007). Drug use and desistance processes. Criminology.

[CR63] Skevington SM, Lotfy M, O'Connell KA (2004). The World Health Organization's WHOQOL-BREF quality of life assessment: Psychometric properties and results of the international field trial. A report from the WHOQOL group. Quality of Life Research.

[CR64] Solis JM, Shadur JM, Burns AR, Hussong AM (2012). Understanding the diverse needs of children whose parents abuse substances. Current Drug Abuse Reviews.

[CR65] Strang J, Gossop M, Heuston J, Green J, Whiteley C, Maden A (2006). Persistence of drug use during imprisonment: Relationship of drug type, recency of use and severity of dependence to use of heroin, cocaine and amphetamine in prison. Addiction.

[CR66] Stuart GL, Temple JR, Follansbee KW, Bucossi MM, Hellmuth JC, Moore TM (2008). The role of drug use in a conceptual model of intimate partner violence in men and women arrested for domestic violence. Psychology of Addictive Behaviors.

[CR67] Urbaniak, G., Plous, S. (2011). R*esearch randomizer (version 3.0)**[Computer software]*. Retrieved on April 22, 2011. New England: Social Psychology Network.

[CR68] Ward J, Davies G, Dugdale S, Elison S, Bijral P (2017). Achieving digital health sustainability: Breaking free and CGL. International Journal of Health Governance.

[CR69] Weekes, J., Moser, A., & Langevin, C. (1999). Assessing substance abusing offenders for treatment. In E. Latessa (Ed.), *Strategic solutions: The international community corrections association examines substance abuse*. Lanham: American Correctional Association.

[CR70] Williams, C., & Chellingsworth, M. (2010). *CBT: A Clinician's guide to using the five areas approach*. London: Hodder Arnold.

[CR71] Wilson IM, Graham K, Taft A (2017). Living the cycle of drinking and violence: A qualitative study of women's experience of alcohol-related intimate partner violence. Drug and Alcohol Review.

[CR72] Young S, Wells J, Gudjonsson G (2011). Predictors of offending among prisoners: The role of attention-deficit hyperactivity disorder and substance use. Journal of Psychopharmacology.

