# Supplementary material for: Protocol for a randomized controlled trial of the Breaking Free Online Health and Justice program for substance misuse in prison settings
Source: Health Justice. 2018 Nov 3;6:20. doi: 10.1186/s40352-018-0078-1 (PMC6755620; doi:10.1186/s40352-018-0078-1)
Supplement: Supplementary file 1 — Appendix A-C. (DOC 36 kb) [file 40352_2018_78_MOESM1_ESM.doc]

**Appendix A: Additional socio-demographic questions**

1. **Date of birth**
2. What is your marital status?

Married / civil partnership

Single

Divorced / separated?

Widowed

Living with partner

1. What was your occupation before you came to prison?

In full-time employment (specify)

In part-time employment (specify)

In full-time education (specify)

In part-time education (specify)

Full-time carer

Unemployed

Other (specify)

1. At what level did you finish full-time education?

Primary school

Secondary school

Further education (college)

Higher education (university)

No formal education

Other (specify)

Appendix B: Standard treatment questions

1. Which of the following treatments for your drug or alcohol difficulties did you receive whilst during your participating in the study?

Prescribed substitute medication

Medication for detox

Mental health medication e.g. antidepressants

One to one key-working

Structured group intervention delivered by practitioners

Mutual aid group work e.g. AA, NA, SMART

Complementary therapies, e.g. acupuncture

1. What kind of practitioners have provided you with support during your participating in the study?

Recovery workers/ drug workers

Medical professional e.g. Psychiatrist, GP

Nurse/ Mental health nurse

Psychologist/ counsellor

Offender manager

Listeners/ peer mentors

1. How often did you receive support from practitioners during your participation in the study?

Every day

More than one day each week

One day each week

Less than one day each week

Once a month

Less than once a month

Appendix C: Reoffending questions

1. Have you been re-arrested since completing your last prison sentence?
2. Have you had attended any more court appearances related to offenses you have been accused of committing since completing your last prison sentence?
3. Have you been convicted of any new offenses since you completed your last prison sentence?
4. Have you been recalled to prison since completing your last prison sentence?
